# Exploring the burden of paediatric acute otitis media with discharge in the UK: a qualitative study

**DOI:** 10.1136/bmjpo-2024-003012

**Published:** 2024-10-21

**Authors:** Elliot Heward, Judith Lunn, James Birkenshaw-Dempsey, John Molloy, Rachel Isba, Darren M Ashcroft, Alastair D Hay, Jaya R Nichani, Iain A Bruce

**Affiliations:** 1 Division of Infection, Immunity and Respiratory Medicine, School of Biological Sciences, The University of Manchester, Manchester, UK; 2 Royal Manchester Children's Hospital, Manchester, UK; 3 Lancaster Medical School, Lancaster University, Lancaster, UK; 4 Alder Hey Children's NHS Foundation Trust, Liverpool, UK; 5 Division of Pharmacy & Optometry, School of Health Sciences, The University of Manchester, Manchester, UK; 6 NIHR Greater Manchester Patient Safety Research Collaboration (PSRC), The University of Manchester, Manchester, UK; 7 Centre for Academic Primary Care, Bristol Medical School, Population Health Sciences, University of Bristol, Bristol, UK

**Keywords:** Child Health, Qualitative research, Adolescent Health

## Abstract

**Background:**

Acute otitis media with discharge (AOMd) results from a tympanic membrane perforation secondary to a middle ear infection. Currently, the impact of AOMd on children and young people (CYP) and their families is not well understood. There is also a need to explore the experience of healthcare professionals in treating AOMd. Interviews with CYP and their parents, and focus groups with medical professionals, were conducted to explore these objectives.

**Methods:**

A total of 26 parents of CYP (age range: 7 months to 15 years) with a history of AOMd (within the last year) and 28 medical professionals were recruited across the UK between August 2023 and March 2024. Healthcare professionals were from primary care (n=17), ear, nose and throat (ENT) (n=7) and emergency medicine (n=4) backgrounds. Thematic analysis was performed independently by three reviewers.

**Results:**

The majority of CYP (n=25/26) (96.2%) had suffered from multiple episodes of AOMd. AOMd has a physical, psychological, educational, financial and social impact on CYP and their parents. Parents found accessing healthcare services and information difficult, which increased parental anxiety. Antibiotic overuse was also a concern among parents. The majority of general practitioners and emergency care staff described using oral amoxicillin, compared with ENT doctors who predominantly prescribed topical antibiotics.

**Conclusions:**

AOMd has a significant impact on CYP and their parent’s daily lives. Need for clear, easily accessible patient information was identified as a priority by the parents of CYP with AOMd. Evidence-based management guidelines should be developed once high-quality evidence is available.

**Trial registration number:**

ISCTRN43760.

**WHAT IS ALREADY KNOWN ON THIS TOPIC**

- Paediatric presentations of acute otitis media with discharge (AOMd) account for approximately 41 000 primary care appointments in the UK each year. Currently, the impact of AOMd on children and young people (CYP) and their families is not understood.

**WHAT THIS STUDY ADDS**

- This study demonstrates the significant impact AOMd has on CYP and their parents’ daily lives. It identifies that patient information material is required to help inform service users. Paediatric AOMd is primarily treated with antibiotics, the type and route are variable.

**HOW THIS STUDY MIGHT AFFECT RESEARCH, PRACTICE OR POLICY**

- This study has collected views from parents and medical professionals on how a future randomised controlled trial should be designed. Future work should aim to create patient information material and develop standardised management guidelines for paediatric AOMd.

## Introduction

 Acute otitis media with discharge (AOMd) results from a tympanic membrane perforation secondary to acute otitis media (AOM), which is one of the most common paediatric infections. Approximately 15% of children and young people (CYP) with AOM develop ear discharge.^1^ CYP with AOMd have been shown to have more severe systemic illness and more disease-related complications, compared with CYP with AOM.^1^ Patient and parental experience has been investigated within the context of AOM but not AOMd.^2^ Approximately 41 000 primary care appointments are required each year to manage AOMd in the UK.^3^ It is essential to explore the impact of AOMd on CYP and their parents to understand the burden of this disease.

The medical management of AOMd in primary and secondary care is heterogeneous, with a mix of oral, topical or no antibiotics being prescribed.^3^ The National Institute for Health and Care Excellence (NICE) recommends that CYP with AOMd be treated with oral amoxicillin.^4^ This recommendation is based on evidence from a subgroup analysis of 116 CYP with AOMd treated with oral antibiotics against placebo, from a meta-analysis of 6 studies.^5^ Topical antibiotics treat the source of the AOMd infection, but there is concern over the potential ototoxic effects which have been addressed by an ear, nose and throat (ENT) UK consensus report.^6^ Understanding the principles governing management strategies from the perspective of different healthcare professionals is vital.

To help standardise medical management for AOMd, high-quality, randomised controlled trials (RCTs) are required. Caldwell *et al* assessed parental attitudes to the participation of children in RCTs, highlighting risk–benefit decision making.^7^ To ensure the design of a future RCT is relevant and impactful, we must engage with the key stakeholders (CYP, parents and medical professionals) to hear their thoughts.

The primary aim was to explore the impact of AOMd on CYP and their parents’ daily lives and to understand medical professionals’ experiences and treatment strategies. The secondary aim was to assess parental and medical professional opinions on how to best design an RCT comparing antibiotic treatments for AOMd.

## Methods

This study follows the Consolidated criteria for Reporting Qualitative research checklist (online supplemental material). The study protocol was registered (ISCTRN43760) and published online.^8^ A research design service (RDS) public involvement grant (RDSNW3687) allowed the involvement of patients and the public at an early stage to help determine the research questions and design. A public involvement group met at regular intervals during the study to help design participant information, refine the interview guide and interpret findings.

A direct-to-participant recruitment method was used by openly advertising the study in medical institutions, charitable organisations and on social media. Interested participants contacted the research team to take part.

### Participants

26 semistructured interviews were performed with parents. Three CYP, over the age of 5 years, joined their parent for the interview. Eligible CYP were under 17 years of age and had experienced at least one episode of AOMd within the 12 months preceding the interview. Six focus groups were conducted with a total of 28 medical professionals who managed CYP with AOMd in daily practice.

### Interview platform and topics

Interviews and focus groups were conducted by JL or EH from 8 August 2023 to 28 March 2024 on Microsoft Teams or telephone, depending on the preference of the participant. Remote consent was given by all parents and medical professionals, and assent from age-appropriate CYP. Interviews and focus groups lasted approximately 45 and 60 min, respectively. The schedule of topics in the interviews and focus groups is provided in online supplemental material. Core topics used by Meherali *et al*, who investigated parental experience of AOM, and Caldwell *et al*, who investigated parental attitudes to participation in RCTs, were adapted for use in this study.^2 7^

### Analysis

Interviews and focus groups were transcribed verbatim onto Microsoft Excel. Transcripts were not returned to participants for comment. A deductive approach to thematic analysis was performed by three independent reviewers (EH, JL and JB-D). Themes from the prior two studies and additional themes that emerged from the interview and focus group data were used. Transcripts of interviews were coded in line with the themes identified.

Parents of CYP were asked to rank order symptoms which was dealt with as quantitative data. Friedman K samples test was performed on the mean ranks of the ranked patient symptoms. Alpha significance value was set at <0.05.

### Patient and public involvement

A patient and public involvement (PPI) group, with lived experience, was created to help determine the most important research priorities. The PPI group met monthly prior to, during and after this study. The PPI group helped to shape the structure and content of the semistructured interviews and patient information material. The PPI group designed material to advertise the study to service users. The results have been interpreted with input from the PPI group. Members of the PPI group have been sent a lay summary and an infographic outlining the results of this research.

## Results

Overall, 26 parents of CYP, 3 CYP and 28 medical professionals took part in this study. Demographics are outlined in table 1. Of the 17 general practitioners (GPs), 14 worked in different practices. All ENT doctors worked at different hospitals; one consultant worked in a tertiary paediatric unit. Both emergency department nurse consultants worked in the same hospital, the registrars worked in different units.

**Table 1:** Patient and medical professional demographics

Demographic	
Children and young people (n=26)	
Female: male	n=10 (38.5%): n=16 (61.5%)
Age	5.3 years (range: 7 months to 15 years)
Medical professional (n=28)	
General practitioner (consultant)	n=15
General practitioner (registrar)	n=2
Ear, nose and throat (consultant)	n=2
Ear, nose and throat (registrar)	n=5
Ear, nose and throat (advanced nurse practitioner)	n=2
Emergency department (nurse consultant)	n=2
Emergency department (registrar)	n=2

### Parent, CYP

#### Frequency of AOMd

All parents except one (n=25/26) described that their child experienced multiple episodes of AOMd over a period of years. Several parents said their child’s ear infections started before the age of one and continued for many years. Infections would occur frequently throughout the year (n=23/26). ‘*He’s suffered from approximately six months old, he’s five years old now and he’s still suffering’ (parent 8). ‘*H*e had seven bouts of infections like seven lots of antibiotics throughout the year’ (parent 24*).

#### Symptoms of AOMd

Parents ranked pain as the most prominent feature of AOMd (n=11/23). There was a significant difference between the ranked symptoms (p<0.01) (table 2). Sleep disturbance and the impact on hearing was frequently discussed by parents.

**Table 2:** Frequency of most important symptom*

Symptom	Mean ranking score	SD	Range of ranking scores
Pain	2.96	2.81	1–4
Difficulty sleeping	4.00	2.74	1–8
Crying	4.96	2.74	2–7
Reduced hearing	5.00	2.81	1–7
Smelly fluid	5.38	2.95	1–8
Colour of fluid	5.54	2.75	1–8
Fever	6.00	2.71	1–8
Appetite	6.62	2.29	2–8

* A symptom ranking of 1 is the worst symptom (range 1–8).

Parents found it challenging to determine the main problem for children who are too young to express themselves. ‘*She experiences smelly fluid and difficulty hearing’ (parent 6). ‘They can’t communicate so you don’t really know what’s wrong’ (parent 26). ‘I wasn’t drinking, I wasn’t eating, I couldn’t sleep’ (child 2*). Two parents highlighted additional challenges for their child with additional communication needs. ‘*He’s also autistic as well so as in having leaky ears you get the aggressive behaviour’ (parent 8*).

There was a theme that once the discharge started, parents would notice an improvement in pain and fever. ‘*It’s like she gets a temperature and then once it bursts, the temperature goes after about 48 hours’ (parent 19*).

### Experiences and effect on quality of life

Parents explained that there was significant parental concern for their child’s ‘suffering’ during an episode of AOMd. Many felt helpless. ‘*As parents it is quite distressing for us seeing this because we are the parent, you are affected knowing that your child is suffering with this’ (parent 12). ‘I'm watching him bang his head on the floor because he can't verbally tell me what hurts’ (parent 1). ‘As parents its really difficult not being able to do a great deal to take that pain away’ (parent 23*). Parents spent considerable time attending doctors’ appointments and presenting to the emergency department, which in turn had an negative impact on their ability to work.

Parents noted hearing loss during and after infections. Many were concerned about long-term implications. ‘*What damage is the infection going to be causing to his eardrums and the affect that that was having on his speech and language development’ (parent 9*). CYP were also stigmatised due to the discharge. ‘*It obviously embarrasses my daughter cause she’s 11 and obviously she gets hearing loss with it as well’ (parent 18*).

### Parents’ concerns and information needs

Many parents found it difficult to find clear or consistent information about the condition. Many researched treatment options online or were advised by family members with lived experience. ‘*I found it hard getting different information from different GPs and then kind of having to go away and do my own research. I think it would have been good if there was more like some uniform care’ (parent 26*).

Dietary changes were frequently discussed by parents. Many were unsure what had caused the ear discharge. ‘*[I was] told by a doctor to come off dairy products and trial that for at least six weeks and see what happens. I thought he had caught [it] from somebody at nursery’ (parent 5*). ‘*[I thought] it was something related to genetics’ (parent 6*).

Antibiotic overuse was a concern among many parents. ‘*I’m definitely getting to the point now where I don’t want him to be on antibiotics much more’ (parent 7*).

### Treatment access and expectations

Healthcare access, exposure to multiple treatment strategies and conflicting advice were key issues discussed. Some felt like their concerns were not taken seriously by medical professionals. It was common that CYP had tried multiple courses of antibiotics and received contradictory advice from medical professionals. ‘*We’ve had liquid, we’ve had sprays, we’ve had drops’ (parent 8). ‘Obviously, seeing him unwell isn’t nice, but it was a lot of going back and to the doctors and taking a while to get referred to the ENT specialists’ (parent 9*).

Most parents expected the infection to improve within 7 days of treatment. Parents frequently used the amount of ear discharge to determine treatment success. ‘*[Expected duration] probably within like three to five days, normally the course of the antibiotics it’s gone’ (participant 7*).

### Views on participation in an RCT

Parents were asked about barriers and motivators which would influence their involvement in a future trial investigating the best management of AOMd. Generally, parents wanted trial involvement to fit around work and their busy lives. ‘*They need to make it quite straight forward, not to complicate it’ (parent 3*). There was no particular preference for oral or topical antibiotic. The majority of parents were against taking part in a trial involving a placebo. ‘*I feel comfortable with my daughter taking part in a clinical trial that really is effective to her condition and not be the dummy medication’ (parent 6*)

### Medical professionals

#### Experiences of managing AOMd

There was acknowledgement that AOMd can be a challenging condition to manage and the impact it can have on the CYP was discussed in all focus groups. ‘*They would recurrently come back to me, which was very frustrating but what you also see is that impact on school, on hearing, on socialising, if it’s a young child on development of speech, interaction’ (medical professional 20*). ‘*It smells terrible, the kid’s not happy, they're not doing well in school. So, then they can't go to swimming class’ (medical professional 5*).

There was a strong feeling that parental concern was a key factor during consultations. ‘*They generally want to see a doctor as soon as possible, to get treatment as soon as possible and to see improvements in their children as soon as possible’ (medical professional 11*).

#### Antibiotic management strategies

There were heterogeneous management strategies depending on the work setting. The majority of GPs and emergency care staff used oral amoxicillin, compared with ENT specialists who use predominantly topical ciprofloxacin. Oral amoxicillin was favoured most frequently for a treatment duration of 5 days. ‘*We always get confused about what to do, it’s only been a few weeks back, I was looking at the guidance to see if you give them antibiotics’ (medical professional 27*).

All GPs referred to NICE guidance to support their management decisions. ‘*There is the NICE guideline that you can, that you can kind of lean on’ (medical professional 21*). Emergency care doctors used local antimicrobial guidelines. ENT doctors had no guidelines to support their management.

#### Non-antibiotic management strategies

All medical professionals agreed that water avoidance was required to prevent repeat infections. There was no consensus on the duration of avoidance required. One GP would see the patient back in 4–6 weeks to check tympanic membrane patency before advising normal activities. Some clinicians advised avoiding using cotton buds in the external auditory canal. The use of topical swabs was varied. Most secondary care professionals would take a swab if not yet taken in primary care.

#### Treatment expectations

Clinical improvement was expected from 5 to 14 days post-treatment commencement. The majority of medical professionals defined treatment success as a reduction or complete cessation of discharge; others used pain reduction as an indicator. ‘*I'll be aiming for total resolution. I think if there was still discharging after a week of antibiotics, I want to probably take a look at them again and reassess things’ (medical professional 6*).

#### Future trial factors

All medical professionals described that they would not recruit CYP with AOMd into a trial with a placebo arm. ‘*I think you’d be hard pressed to justify a placebo in that situation’ (medical professional 18*). They would also be against recruiting CYP who are systemically unwell to any trial. There was a strong opinion that a trial design should mirror current practice and clear patient information is important to help parents understand the trial.

## Discussion

These findings show the physical, psychological, education, financial and social impact of AOMd on CYP and their parents and the heterogeneity of treatment provided in the UK. Our results demonstrate that the quality-of-life impact of AOMd on CYP and their parents is similar to those with recurrent AOM.^2^ The findings differ considerably when comparing information needs and treatment experience. Parents of CYP with AOMd found it difficult to ascertain information about the condition while parents of CYP with AOM, based in the USA, found adequate information from healthcare providers or online.^2^ In this study, parents repeatedly commented on the variation and frequency of antibiotic treatment for AOMd which was not reported in the AOM group.^2^

AOM causes otalgia by stretching of the tympanic membrane and irritation of cranial nerves passing through the temporal bone.^9^ Once the tympanic membrane perforates and pressure is released, the assumption is that otalgia and fever reduce. Smith *et al* demonstrated that otalgia was more frequently associated with AOM (56%) compared with AOMd (11%).^1^ However, participants in this study rated otalgia as the worst feature. This could be due to overlapping symptoms experienced from AOM to AOMd or ongoing noxious effects of the infection such as the development of otitis externa secondary to discharge. Other highly ranked symptoms such as difficulty sleeping and crying may well be associated with pain levels.

Our results demonstrate there is a significant financial and psychological impact on parents. Numerous healthcare appointments require parents to take time off work in addition to the travel expense. The psychological burden on parents has been shown when caring for chronic childhood illness but there is little evidence in recurrent acute disease such as AOMd.^10^ Another key problem identified in this disease context is the lack of patient information which contributes to parental anxiety. Limited medical information for AOMd likely prevents medical professionals from providing adequate counselling to parents. There is a need for patient, parent and professional-level information for AOMd.

Antimicrobial resistance is a growing global problem.^11^ The majority (72%–92%) of CYP with AOMd receive antibiotics despite there being minimal evidence for their effectiveness.^1 3^ Current NICE guidance within the UK recommends treatment with oral amoxicillin for CYP with AOMd.^4^ However, a recent study has shown that topical antibiotic treatment is more effective than oral antibiotics for tympanostomy tube otorrhoea.^12^ Clinicians from both primary care and emergency departments favour oral antibiotics. ENT doctors prefer topical antibiotics. Despite working in secondary care, ENT doctors also manage acute presentations of AOMd in follow-up and rapid access clinics, emergency departments and provide advice to other specialities. The difference in prescribing practice is likely multifactorial, reasons may include their familiarity and access to topical antibiotic drops. Treatment variation and antibiotic overuse were a key concern of parents, with CYP frequently receiving multiple courses of various types of antibiotics. Parental concern regarding antibiotic overuse in middle ear infections has also been reported in North America.^13^ High-quality evidence is required to identify the most effective antibiotic treatment for AOMd.

The use of appropriate outcome measures in healthcare research is essential to derive meaningful results. Both parents and medical professionals most commonly use cessation of discharge as an indicator for treatment success. However, the expected time frame for cessation is variable from 5 to 14 days. Parental attitudes to participation in a future RCT were comparable to the findings of Caldwell *et al* in that the benefits of taking part should outweigh the risks.^7^ Parents and medical professionals are cautious of participation in trials involving placebos as they find it difficult to justify when treating a paediatric infection.

The strength of this study is the large sample of participants from across the UK with the combination of perspectives from both CYP, parents and medical professionals. The majority of medical professionals were based at different departments which provides an overview of UK practice. Data saturation was met in both groups. The main limitation of this study is a recruitment bias in that parents self-identified themselves to be recruited. It is likely that those CYP who frequently suffer would be more likely to be volunteered by their parents to participate, skewing the cohort to those more severely affected by AOMd. This research did not address the role of socioeconomic disparities in this disease context. This is a requirement for further research.

This research highlights that AOMd has a significant physical, psychological, educational, financial and social impact on CYP and their parents. There is an urgent need for tailored patient information materials in this disease context. Treatments for AOMd vary depending on the clinician setting. To standardise management high-quality evidence is required to support treatment guidelines for paediatric AOMd. Based on the results presented here, such a trial should be patient-centred to work around families’ busy lives and be codesigned by service users.

## supplementary material

10.1136/bmjpo-2024-003012 online supplemental material 1

## Data Availability

All data relevant to the study are included in the article or uploaded as online supplemental information.
