# Supplementary material for: Exploring the burden of paediatric acute otitis media with discharge in the UK: a qualitative study
Source: BMJ Paediatr Open. 2024 Oct 21;8(1):e003012. doi: 10.1136/bmjpo-2024-003012 (PMC11499759; doi:10.1136/bmjpo-2024-003012)
Supplement: online supplemental material 1 [file bmjpo-8-1-s001.pdf]

**Supplementary Material 1. Interview Schedule**

**Families and Carers and Children / Young People with Acute Otitis Media with Discharge**

---

**Welcome and introduction - Seek verbal consent to continue, reminder of audio-recording of interview (/video recording if on Teams), re-cap of project and plan for interview. Perform member check after each question or where necessary.**

*The study is aiming to understand what it is like for children and young people to live with an ear infection which causes a leaky ear. The interviews will explore your views and we are interested in your experience. It will help us to understand more about children and families' experiences of leaky ears. We also wanted to talk to you about how we might conduct research in the future on leaky ears, and if you think it is acceptable way of doing things. With this information we hope to help change how we treat children and young people with leaky ears.*

*If you do not feel you are able to comment on any area, please say so and we can stop at any time. Do you have any questions before we start?*

1. Can you tell me when you had leaky ears and for how long?

2. Can you tell me about the impact of having leaky ears has had on you / your child's everyday life?
    - Prompt: What we mean is has it stopped you from doing things that you were doing before or changed the way you feel about things. If it has, can you tell me more about it.
    - Prompt: Please tell me about any other things that also had an impact. Impact on school, sport, social, mental wellbeing

3. Which aspect about the leaky ears affected you the most?
    - Prompt: What we mean is things like the smelly fluid from the ear, pain in the ear, fever, crying, the need to visit the doctors, or problems with sleeping?

4. Can you put these in order, symptom that most affected to least affected you:
    - Smelly fluid, colour of fluid, pain, fever, crying, difficulty sleeping, reduced hearing and appetite

5. Can you tell me about your experience of being treated for leaky ears from when it first started up until now?
    - Prompt: Can you tell me about what happened when you first noticed leaky ears and asked for help. Then what happened after this?

6. Can you tell me about any treatments you were given for the leaky ears?
    - Prompt: Can you tell me about any liquid/tablet antibiotics or ear drops you were given to use by the doctor and was there a reason ever to stop early
    - What was your experience giving the treatment and which was preferred?
    - Did you try/use any other ways to treat your leaky ears? Where did you obtain this information/advice?

7. What do you think could be done better or differently to improve the way patients with leaky ears are treated in the future?

8. How long after treatment was started would you expect the symptoms to improve?

9. How long would you be happy to give your child an antibiotic medicine to treat the leaky ear?

10. What would be the most important sign that the infection is improving?

11. How much improvement in the 'most important sign' would show the treatment was successful?

12. How long would you expect the treatment to take to improve the most important sign?

13. If we offered you 2 different treatment options what difference in time to the 'most important sign' resolution would make you make you pick one over the other?

*I would like to ask you a few more questions about taking part in research in the future. This is not an invitation to take part in another study now but more general questions about taking part in research studies.*

Randomised Controlled Trials

14. What do you know about research studies that try to decide which treatment is the best called randomized controlled trials?
    - Prompt: (An explanation of randomized controlled trial is given by facilitator): "*A randomised controlled trial is a special study which can find out what is the best treatment, when it is not clear which treatment is best. When a randomised controlled trial is conducted, nobody, not even the doctors or scientists, know which is the better treatment.*
      *There are usually two treatments being compared, and the treatment each child receives is decided by chance. It is necessary to decide the treatment by chance to make sure that at the beginning of the study, both groups of children are very similar. Therefore, we know that any differences at the end of the study will be due to the treatment alone."*

15. What questions might you have if your child with a leaky ear was invited to participate in a trial where their treatment was randomly selected?

16. If the options were to have a liquid antibiotic orally or antibiotic drops to the ear, how would you feel about the child's treatment being chosen by chance to one medicine or another?
    - Prompt: Do you have any feelings about giving oral liquid antibiotics or antibiotics in drop form to the ears

17. How do you feel about the child's treatment being allocated by chance which could be a placebo (which is a dummy medicine)?
    - Prompt: Sometimes, randomised controlled trials involve the use of a placebo, which is a nonactive medication. It is used when testing whether using a particular treatment is better than not using anything.

Motivators and Barriers

18. What would make you more likely to get involved with an RCT?
    - Examples - improve treatment for children/ get better care for their child (better treatment compared to available or access to specialist doctors quicker)

19. What would put you off joining an RCT?
    - Examples - extra visits, safety of medications

20. How would you feel if you went to your GP and were offered to sign up to a study on the same day? Would you want time to think about it?

21. If we gave you a diary to fill in for a week every day, would you prefer a paper diary or one online?

22. What do you think are some of the things that researchers need to think of when they design research for leaky ears?

**Anything not covered?**

Is there anything that we haven't covered in the interview that you think we should know or think about?

**Closing and thanks -** Thank for their time and contribution.

**Clinicians (nurses, audiologists, allied medical professionals, GPs, Emergency Department and Otolaryngology doctors).**

---

**Welcome and introduction - Seek verbal consent to continue, and check that all members must interact with patients aged 16 and below with paediatric otorrhoea in daily practice to be eligible to take part. Provide a reminder of the video-recording of focus group (Teams) will be saved, re-cap of project and plan for focus group discussion. Perform member check after each question or where necessary.**

*The study is aiming to understand how and why children and young people (CYP) with acute otitis media with discharge are treated in primary and secondary care and to understand what treatments are acceptable to medical professionals. We are interested in your perceptions, based on your knowledge and experience. If you do not feel you are able to comment on any area, please say so. Do you have any questions before we start?*

1. Could everyone please firstly introduce themselves, provide their job role and say roughly how regularly they see children with otorrhoea.
    - Prompt: On average, how many children with otorrhoea would you see in a month?

2. What are your experiences of managing CYP with otorrhoea?
    - Impact on patient, their QoL/communication
    - Number of healthcare attendances
    - Recurrent condition or not
    - Straightforward/challenging to manage
    - Complications of condition

3. Please describe how otorrhoea in a 3, 6 and 14 year old patient is normally treated and what are the reasons behind management decisions.
    - When do you treat them? first presentation / future presentation / significant symptoms / delayed antibiotic course?

- How do you treat children? Oral / topical antibiotics, analgesia, advice given other treatments like removal of otorrhoea
- What is a typical treatment duration?
- What is your second line treatment if there’s no or limited improvement? Longer course or change in treatment
- When would you refer the patient onwards (if working in primary care)
- Why do they treat in this way? Experience / normal practice / current guidelines
- Why do you decide to treat – pain, fever, otorrhoea, hearing loss, prevent complications, age, co-morbidities, vaccination status
- Do you swab the discharge? Wait for results to help treatment?

4. Do you give any lifestyle advice? (examples: Swimming avoidance, water precautions, Time off daycare, exposure to passive smoking/pets)

5. Do you feel supported with clear guidelines or evidence how to manage this condition?

6. Routinely how long would you prescribe antibiotics for in one course?

7. From your perspective, how would you feel recruiting patients to an RCT with a placebo alone option, placebo with an active treatment? Are there some patients you wouldn’t want to give a placebo?

8. How long after treatment was started would you expect the symptoms to improve?

9. What would be the most important sign that the infection is improving?

10. What is the least you would expect from the treatment to indicate its working in terms of the most important sign? - What is the minimum improvement in the 'most important sign' you would regard as showing the treatment was successful?

11. If you prescribed two treatments what is the minimum difference in the 'most important sign' that showed one was better than the other? What would make you pick one treatment over another treatment.

12. If you prescribed two treatments what is the maximum difference in the 'most important sign' you would regard as showing one was equivalent to the other?

I would like to ask you a few more questions about taking part in research in the future.

13. What would improve participation in such a study in Primary Care?

14. What factors would be a barrier to participation?

15. For people working in primary care: how do you feel about patients being identified in primary care then referred to a central research team for randomisation and treatment?

16. From your perspective, how would you feel about your patients receiving either oral or topical antibiotic treatment in an RCT?

17. If a RCT showed that a different antibiotic type or route was more effective than your current preference would you change practice?

**Anything not covered?**

Is there anything that we haven't covered in the focus group that you think we should know or think about?

**Closing and thanks -** Thank for their time and contribution.
