# Supplementary material for: Exploring the burden of paediatric acute otitis media with discharge in the UK: a qualitative study
Source: BMJ Paediatr Open. 2024 Oct 21;8(1):e003012. doi: 10.1136/bmjpo-2024-003012 (PMC11499759; doi:10.1136/bmjpo-2024-003012)
Supplement: Supplementary file 2 [file bmjpo-2024-003012.reviewer_comments.pdf]

PEER REVIEW HISTORY

BMJ Paediatrics Open publishes all reviews undertaken for accepted manuscripts. Reviewers are asked to complete a checklist review form and are provided with free text boxes to elaborate on their assessment. These free text comments are reproduced below.

ARTICLE DETAILS

**TITLE (PROVISIONAL)**	Exploring the burden of paediatric acute otitis media with discharge in the UK: a qualitative study
**AUTHORS**	Heward, Elliot; Lunn, Judith; Birkenshaw-Dempsey, James; Molloy, John; Isba, Rachel; Ashcroft, Darren M; Hay, Alastair; Nichani, Jaya R; Bruce, Iain A

VERSION 1 - REVIEW

**REVIEWER NAME**	***John Rocke***
**REVIEWER AFFILIATION**	Bolton NHS Foundation Trust, ENT
**REVIEWER CONFLICT OF INTEREST**	
**DATE REVIEW RETURNED**	03-Sep-2024

**GENERAL COMMENTS**	This study provides a fantastic foundation for future research. The study presents patient, parent and clinical evaluations relating to their experiences concisely. There is a clear need for this type of research in our speciality and this study addresses this gap in knowledge very well.
